# Urbanization’s Impacts on Ecosystem Health Dynamics in the Beijing-Tianjin-Hebei Region, China

**DOI:** 10.3390/ijerph18030918

**Published:** 2021-01-21

**Authors:** Fan Wu, Xiaoke Wang, Yufen Ren

**Affiliations:** Research Center for Eco-Environmental Sciences, Chinese Academy of Sciences, Beijing 100085, China; fanwu_st@rcees.ac.cn (F.W.); wangxk@rcees.ac.cn (X.W.)

**Keywords:** ecosystem health, indicators, urbanization, spatial correlation, sustainable development

## Abstract

Accelerated urbanization disturbs ecosystems and influences ecosystem structures and functions. Ecosystem health (ESH) assessments in regions undergoing the rapid urbanization process assist us in understanding how ESH changes and how urbanization specifically affects ESH. We assessed the ESH of Beijing-Tianjin-Hebei (BTH) region, China, including the ecosystem service value (ESV). In this study, we selected nine indicators and applied multiple pieces of software, including ArcGIS10.4, Fragstats4.2 and Geoda1.14 to detect the impacts of urbanization on regional ESH in 1995, 2000, 2005, 2010 and 2015. The results show that ESH in the BTH region increased from 2000 to 2015, especially in the northern parts. The improvements were due to the implementation of key ecological restoration projects protecting and re-establishing the forest in the north. Furthermore, the spatial correlation results indicate that urbanization had a negative impact on ESH in the BTH region, of which the dominant factor was the population density in 1995. The dominant factor was the construction land proportion from 2005 to 2015. We suggest that ecological restoration projects should be continued in northern regions with strong and relatively strong ESH levels to maintain high-level ecosystem health. In addition, more attention should be paid to the ESH level improvement in peri-urban areas.

## 1. Introduction

Urbanization appears to be an inevitable process in socioeconomic development [1,2,3]. Better education, medical care and other aspects attract more people to urban areas [4]. However, urbanization also causes some economic and environmental problems, such as heavy traffic, noise pollution and decline in biodiversity [5,6]. Urbanization increases the extent and intensity of human activity; it has influenced the structures and functions of ecosystems at unprecedented speed and scale [7,8,9]. Ecosystems are disturbed by urbanization through the creation of construction land, extensive industrialization, large-scale deforestation for farming and other human activities. These great pressures may lead to ecosystem degradation and decreasing ecosystem health [10]. Once the ecosystem is damaged, its ability to provide various resources will be negatively affected, and ecosystem health will decrease [11]. Thus, more attention should be paid to ecosystem health assessment and sustainability of the city.

In 1989, Rapport first discussed what ecosystem health (ESH) is and how to measure ecosystem health [12]. In 1992, Rapport (1992) explained the concept of ecosystem health in detail [13], which defined the health of an ecosystem as its ability to maintain an organizational structure, and to recover after disturbances with self-regulating processes. A healthy ecosystem has an integral structure, has resistance to external disturbances and offers sustainable resources for human beings [12]. Structural and functional integrity of an ecosystem are the basic prerequisites for achieving sustainable development. Based on Rapport’s studies [13], ESH assessment usually involves three indicators: vigor, organization and resilience. First, vigor represents the substances or energy from nutrient cycling and productivity. Second, organization refers to the diversity and quantity of relationships between the components of the ecosystem. It represents the complexity of the ecosystem’s structure. Resilience refers to the ability of the system to gradually recover from external pressure or the capability of the system to resist external interference. This framework has been widely accepted and applied in regional ESH assessments [14]. Lu (2003) [15] calculated the ecosystem health of artificial vegetation in Zhongwei County, Ningxia Province, China based on vigor–organization–resilience (VOR) theory; Xiao (2019) [16] applied a hidden Markov model (HMM) to simulate the internal–external correlations of ecosystem status based on the VOR framework in Shanghai Hangzhou Bay Metropolitan (SHBM); De Toro and Iodice (2018) [17] assessed ecosystem health level of the metropolitan area of Naples by integrating geospatial data. Li (2013) [18] assessed the ecosystem health of the alpine meadow of the Qinghai-Tibetan Plateau, China, by using a modified VOR framework.

An assessment of ecosystem health is an essential way to understand interactions between an ecosystem and human activity. Additionally, it provides information about how human beings impact said ecosystem during the urbanization process. However, most ESH assessment studies only chose indicators representing vigor, organization and resilience, but ignore ecosystem function [19]. Ecosystem services, defined as the benefits humans obtain from an ecosystem, reflects the ecosystem’s function [20]. Costanza (2012) [21] proposed that a healthy ecosystem provides kinds of ecosystem services sustainably for humans. In urban areas, a healthy ecosystem provides resources and products to meet the needs of urban residents [22]. It not only creates and maintains the environmental conditions necessary for human survival; but also provides food, water, medicine and other production or materials for human beings. Moreover, it provides recreation, leisure and aesthetic enjoyment for human beings [23,24]. This reflects the satisfaction of human needs by the ecosystem, or benefits to human survival and life [25]. Ecosystem health refers to being able to maintain a stable structure and ecological processes; adjust and recover from external disturbances; and ensure the supply of sustainable and satisfactory ecosystem services [26,27]. Since the demands of urban residents on the ecosystem is increasing, the current ecosystem health assessment should pay more attention to the benefits of natural ecosystems to humans. Yuan (2019) [28] assessed ESH of Guangzhou City, China based on VOR framework and the ecosystem services involved in it. Peng [29] analyzed ESH of Shenzhen City, China, in four aspects: organization, vigor, resilience and regional ecosystem services. This new framework has made a connection between the typical ESH assessment and regional ecosystem services.

In this study, we assessed the ecosystem health dynamics of the Beijing-Tianjin-Hebei (BTH) region, China, based on the “vigor–organization–resilience–ecosystem services” framework in 1995, 2000, 2005, 2010 and 2015 by utilizing the multi-source data and statistics. We also studied the impacts of urbanization on ESH by using Moran’s I index. The objectives of our study were as follow: (1) to measure and map ecosystem health level by integrating land use data and socioeconomic data; (2) to analyze spatio-temporal dynamics of ecosystem health and urbanization; (3) to study the correlations between ESH level and urbanization level from a spatial perspective. Based on assessment of ESH and analysis of urbanization, we hope to clarify how urbanization affects ESH in the BTH region. In addition, we expect this study will help to facilitate the ecosystem-based management of urbanization and further promote sustainable development in urban regions.

## 2. Data and Methods 

### 2.1. Study Area and Data Collection

**Figure 1:**
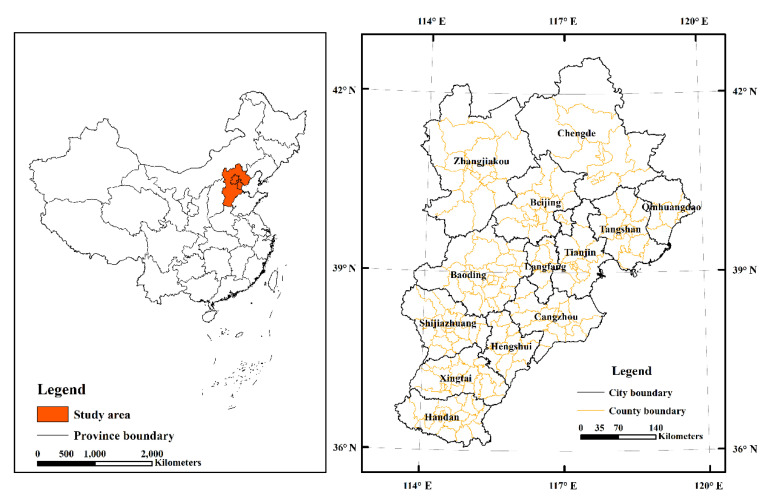
Location of the Beijing-Tianjin-Hebei region.

The Beijing-Tianjin-Hebei (BTH) region is composed of Beijing, Tianjin and Hebei Provinces. Beijing, the capital of the People’s Republic of China, is an international center of culture, politics and economics. Tianjin is the largest port city in northern China and an important transportation center. Hebei Province is located in the North China Plain, with the Bohai Sea in the east, the Taihang Mountain in the west and the Yanshan Mountains in the north (Figure 1). 

The total land area is approximately 216,600 km^2^. The elevation decreases from the northwest to the southeast. The BTH region has a typical temperate, continental monsoon climate with cold winters and hot summers. The annual average temperature is about 3–15 °C and the annual average precipitation is 304–750 mm. Rapid urbanization has occurred in BTH region. In the past fifteen years, the population in urban areas has increased by 0.68 million [30,31,32]. In 2018, the gross domestic product (GDP) was 850 billion yuan, accounting for 9.48% of national GDP [30,31,32]. As China’s “capital economic circle,” Beijing-Tianjin-Hebei is the region with the largest and most vigorous economy in northern China.

The Landsat TM/ETM+ images at a resolution of 30 m from 1995, 2000, 2005, 2010 and 2015 were obtained from the Geospatial Data Cloud (http://www.gscloud.cn/) [33]. After radiation calibration, geometric correction, image enhancement, visual interpretation and supervised classification, we got the land use data at a resolution of 30 m in ENVI 5.1. The kappa coefficients are greater than 0.85. There are 6 land use types: forest, grassland, cropland, built-up land, water and unused land. By using land use data, landscape indices were calculated in Fragstats 4.2 software. The socio-economic data were obtained from yearbooks of BTH in official website: Beijing Municipal Bureau of Statistics (http://tjj.beijing.gov.cn/) [30], Tianjin Municipal Bureau of Statistics (http://stats.tj.gov.cn/) [31] and Hebei Provincial Bureau of Statistics (http://tjj.hebei.gov.cn/) [32]. Normalized difference vegetation index (NDVI) and net primary production (NPP) data were derived from the Resource and Environment Science Data Center (http://www.resdc.cn/) [34].

### 2.2. Ecosystem Health Assessment 

In this study, the ESH of BTH region was assessed from four dimensions according to Costanza’s study [21] and Peng’s study [29]: vigor, organization, resilience and ecosystem service value (ESV). We applied the vigor–organization–resilience–ESV framework to select indicators and assess ESH.

The formula used is as follows [35]:

(1) $$ESH=\sqrt[{4}]{V\times O\times R\times ESV}$$

where ESH represents the ecosystem health; V represents ecosystem vigor; O represents ecosystem organization; R represents ecosystem resilience; ESV represents ecosystem services value. In our study, to detect the dynamics of ESH in BTH from 1995–2015, ESH was divided into five different levels: strong (80–100%), relatively strong (60–80%), ordinary (40–60%), relatively weak (20–40%) and weak (0–20%).

Based on the traditional vigor–organization–resilience framework, vigor represents the ecosystem’s activity or productivity. Vigor is generally quantified by productivity (such as primary productivity, net primary productivity), biomass and metabolic rate. In this study, NDVI was applied to quantify vigor; it has widely been proven to be effective in assessing the primary productivity of vegetation [36]. The higher the NDVI, the higher the vigor. Organization is quantified from two aspects—one is the species diversity of the ecosystem, and the other is the complexity of the ecosystem [37,38]. In this study, we quantified organization from landscape heterogeneity and landscape connectivity by using landscape indices. In general, landscape heterogeneity is measured through landscape diversity and landscape fractal dimension. In our study, we used Shannon’s diversity index (SHDI) and patch density (PD) to quantify the landscape diversity [39]. PD represents the number of patches per unit area [40]. The higher the SHDI value is, the more complex the ecosystem. The area-weighted patch fractal dimension (AWMPFD) was used to quantify the landscape fractal dimension [41]. The higher AWMPFD, the higher the landscape heterogeneity. Ecosystems that are less disturbed by human activities have higher AWMPFD and higher organization [42]. Landscape connectivity was measured using the patch cohesion index (COHESION), contagion index (CONTAG) and integral index of connectivity (IIC) [43] (Table 1). The higher COHESION, CONTAG and IIC are, the higher the landscape connectivity and the higher the organization [44].

For ecosystem resilience, it is influenced by many factors, including climate, vegetation, biodiversity and human activities [45,46]. Here, we considered climate and vegetation to quantify ecosystem resilience.

The formula is as follows:

(2) $$R=\frac{N}{r}$$

(3) $$r=\frac{E}{P}$$

where R represents the resilience value; N represents NPP; r represents the drought index. E represents evaporation; P represents precipitation. The higher the resilience value, the more resilient the ecosystem.

Ecosystem service value (ESV) represents the benefits that humans obtain from an ecosystem [47]. A healthy ecosystem has the ability to supply services for humans [48]. Xie presented ecosystem service value coefficients of six land use types in China based on quantitative surveys [49]. Liu calculated the ecosystem service value in BTH region [50] and Wang revised the ecosystem service value coefficients in BTH region based on Xie’s study [51]. In this study, considering the availability and reliability of ecosystem value coefficients, we referenced these studies to measure ESV based on six land use types. For recreation and aesthetic value of forest and grassland, we referenced Urbis’s study [52] to revise the coefficients.

### 2.3. Urbanization Level 

Urbanization is a multi-dimensional process. It is a process of economic development, population increase, expansion of urban land use and lifestyle change [53]. Economic development is the basis; population increase and urban area expansion are representational performance; and living standard improvement is the final goal [54]. Population growth is a remarkable characteristic of urbanization. Urban regions are centers of population and various economic activities. The growth of an urban population is an objective requirement for the development of an economy, since it brings more labor [55]. Population growth increases the need for construction land for residents and economic activities to support development. From a geographic perspective, urbanization is regarded as a process of transforming rural areas or natural areas into urban areas [56]. 

In this study, we chose people density (PD) (person km^−2^), gross domestic product (GDP) density (yuan km^−2^) and construction land proportion (CLP) to measure urbanization from demographic, economic and geographic perspectives, respectively.

### 2.4. Spatial Correlation 

Moran’s I is an important index for studying relationships from the spatial perspective [57]. Moran’s I is applied abroad in studies on the structure of ecosystems [58], environmental pollution [59] and ecosystem process dynamics [60]. It is divided into two types: the global bivariate Moran’s I and local bivariate Moran’s I (bivariate LISA). The former provides summary statistics for overall spatial clustering. The latter is applied typically to measure the relationships between spatial units and their neighboring spatial units to reveal clustering patterns. In this paper, we utilized two Moran’s I indices to study the influence of urbanization on ESH by analyzing the spatial correlations. The global bivariate Moran’s I was utilized to measure the spatial correlation between urbanization and ESH in the whole area. The local bivariate Moran’s I was utilized to measure the clustering patterns of urbanization and ESH in BTH region [61]:

(4) $$I=\frac{N\sum_{i}^{N}\sum_{j\neq i}^{N}W_{ij}Z_{i}Z_{j}}{\left(N-1\right)\sum_{i}^{N}\sum_{j\neq i}^{N}W_{ij}}$$

(5) $$I_{kl}^{i}=Z_{k}^{i}\sum_{j=1}^{N}W_{ij}Z_{l}^{j}$$

(6) $$Z=\frac{X_{k}^{i}-{\bar{X}}_{k}}{\sigma_{k}}$$

(7) $$Z_{l}^{j}=\frac{X_{l}^{j}-{\bar{X}}_{l}}{\sigma_{l}}$$

where I is the global bivariate Moran’s I for the urbanization level and ESH; $I_{kl}^{i}$ is the local bivariate Moran’s I for the urbanization level and ESH; N is the number of all spatial units in BTH region; $W_{ij}$ represents a weight matrix to measure the spatial correlations between the *i* spatial unit and the *j* spatial unit [62]; $Z_{i}$ is the deviation between the attribute of the *i* spatial unit and the average of the attribute; $Z_{j}$ is the deviation between the attribute of the *j* spatial unit and the average of the attribute; $X_{k}^{i}$ is the value of attribute *k* of spatial unit *i*; ${\bar{X}}_{k}$ is the average of attribute *k*; $\sigma_{k}$ is the variance of attribute *k*. $X_{l}^{j}$ is the value of attribute *l* of spatial unit *j*; ${\bar{X}}_{l}$ is the average of attribute *l*; and $\sigma_{l}$ is the variance of attribute *l*.

The value of I/$I_{kl}^{i}$ ranges from −1 to 1. A positive I/$I_{kl}^{i}$ value represents a positive spatial correlation between urbanization and ESH, which signifies that a unit with a high level of urbanization is surrounded by units with high ESH. A negative I/$I_{kl}^{i}$ indicates a negative spatial correlation—a unit with a high urbanization level is surrounded by units with a low ESH [63]. The higher absolute value of I/$I_{kl}^{i}$ indicates that the spatial correlation is stronger. In order to get reliable results, we set the statistically significant value at the 1% level for the spatial correlation between urbanization and ESH. We calculated the global bivariate Moran’s I and local bivariate Moran’s I in Geoda1.14 software.

## 3. Results

### 3.1. Ecosystem Health Dynamics 

**Figure 2:**
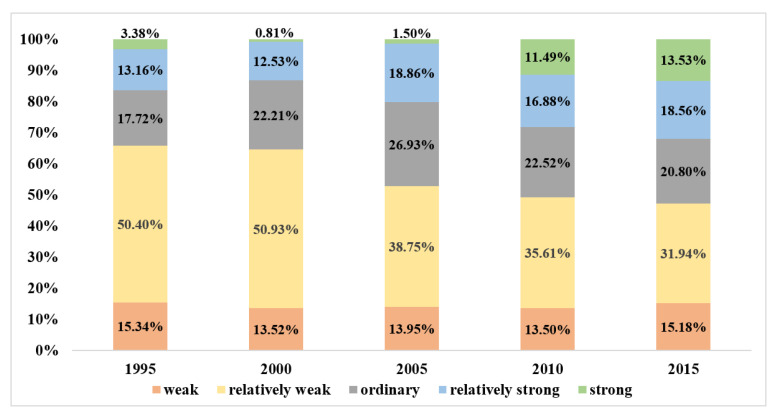
Proportions of areas with different ecosystem health (ESH) levels from 1995 to 2015.

The proportions of areas with five ESH levels changed from 1995 to 2015, as Figure 2 displays.

From 1995 to 2015, the proportion of areas that were weak was stable at 13–15%. The proportion of areas at an ordinary level increased from 17.72% in 1995 to 26.92% in 2005, and then decreased to 20.80% in 2015.

From 1995 to 2015, apparent changes were discovered in the proportions of areas that were relatively weak in ESH and strong in ESH. From 1995 to 2000, areas with relatively weak levels of ESH slightly increased during 1995–2005, and then decreased sharply from 50.93% in 2005 to 31.94% in 2015. Areas with strong levels of ESH occupied only 0.81% of the study area in 2000, with an obvious increase from 1742 km^2^ in 2000 to 29,142 km^2^ in 2015. 

The sum of areas with relatively strong and strong levels of ESH decreased during 1995–2000, and then increased from 13.33% in 2000 to 32.08% in 2015. By contrast, the proportion of the total area with relatively weak ESH and weak ESH decreased from 64.45% in 2000 to 47.12% in 2015. These indicate that the increase of ESH happened mainly between 2000 and 2015. The results indicate that the overall ESH level of BTH improved from 2000 to 2015. The trend of improvement accelerated after 2005.

**Figure 3:**
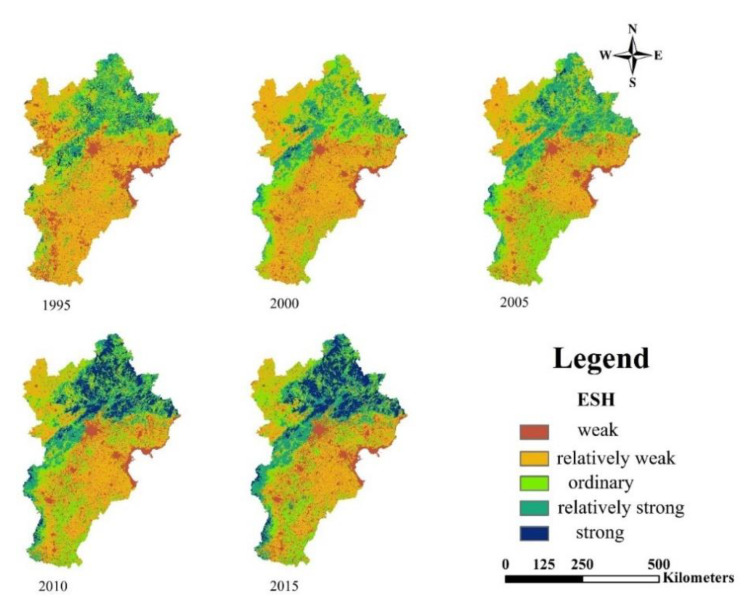
Spatial dynamics of ESH from 1995 to 2015.

The spatial dynamics of ESH in 20 years of BTH region are displayed in Figure 3.

In 1995, regions with weak ESH were mainly distributed around the major cities, ports and parts of the northwest. Regions with relatively weak ESH were distributed in southern and northwest regions of BTH. Regions with ordinary and relatively strong levels of ESH were distributed in the northern part of BTH (Chengde). Regions with strong ESH were rare and distributed in the north.

From 1995 to 2000, ESH in north BTH decreased. Some regions of relatively strong ESH converted into regions of ordinary level. In the northwest and southwest of BTH, some regions of weak ESH converted into regions with relatively weak ESH.

From 2000 to 2015, in the northwest and southern parts of BTH, some regions with relatively weak and weak ESH converted to regions with ordinary and relatively strong ESH. From 2005 to 2015, many regions with strong and relatively strong ESH expanded in north, northwest and west of BTH, becoming a northeast–southwest trend belt.

These results illustrate that ESH level of BTH region fell in 1995–2000 and then improved from 2000 to 2015.

### 3.2. Urbanization Dynamics

**Figure 4:**
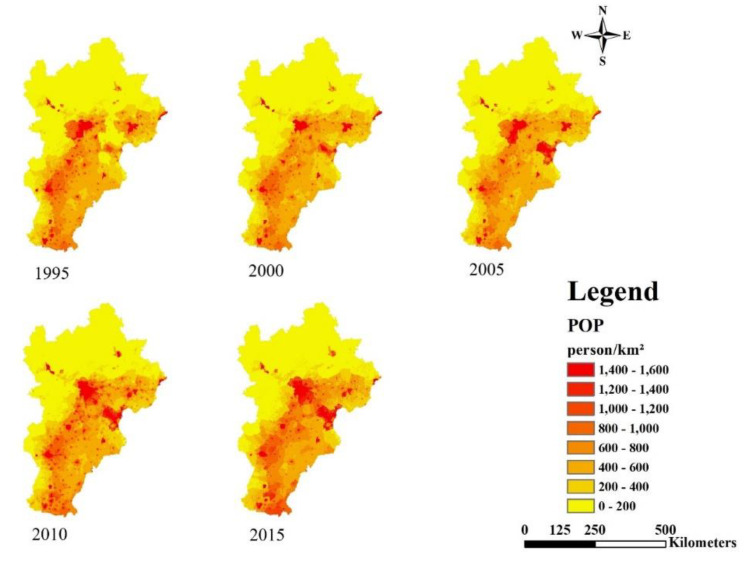
Spatial dynamics of population urbanization (POP) from 1995 to 2015.

In order to study the spatial relationships between urbanization and regional ESH of BTH, we used ArcGIS 10.4 to map urbanization of BTH during 1995 to 2015. As shown in Figure 4, in major cities, the population density increased from 1995 to 2015. High-density population regions expanded from cities to suburbs. 

**Figure 5:**
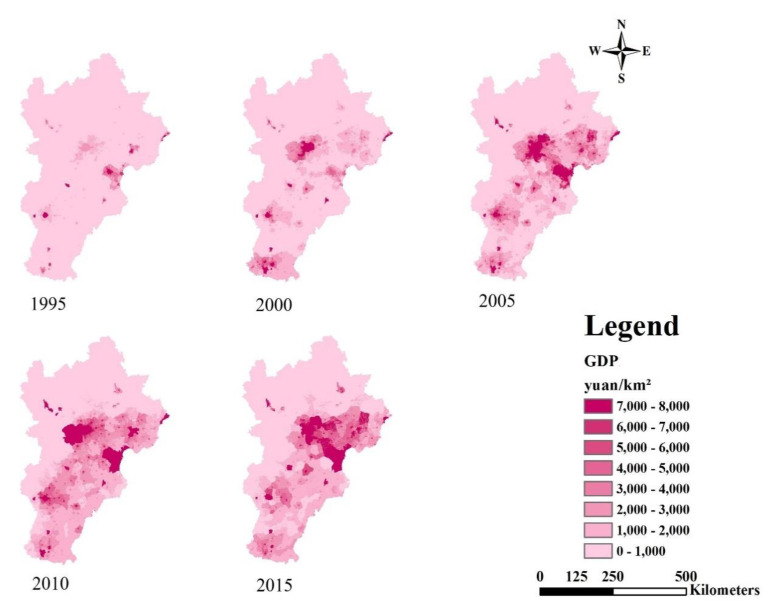
Spatial dynamics of GDP from 1995 to 2015.

As shown in Figure 5, the GDP was high in cities, gradually declining from the major cities to the suburbs. The GDP of cities increased, and this trend was accelerated obviously between 2000 and 2015. Regions with high GDP spread from cities to suburbs.

**Figure 6:**
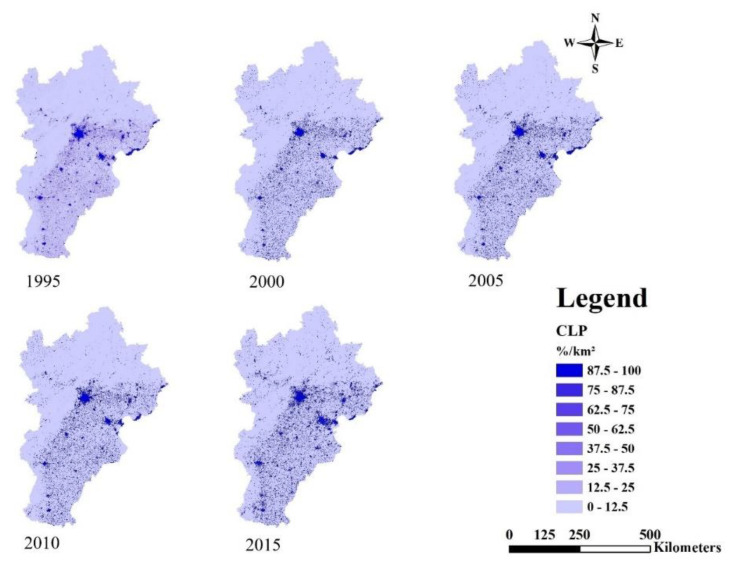
Spatial dynamics of construction land proportion (CLP) from 1995 to 2015.

As shown in Figure 6, from 1995 to 2015, CLP in suburbs increased, especially in the southeast part of BTH. Regions with higher urbanization were found in the middle parts of BTH, especially in Beijing, Tianjin and Tangshan. There is a triangular trend of urbanization development composed of these three cities. The results showed that the urbanization level is lower in northern Beijing-Tianjin-Hebei and it remains stable. Urbanization is spreading from main cities to suburbs and the urbanization level in the southern parts of BTH increased from 1995 to 2015.

### 3.3. Spatial Relationship between ESH and Urbanization

The results of global bivariate Moran’s I demonstrated strong spatial correlations between ESH and urbanization (all Moran’s I values < 0 and *p*-values = 0.01) (Table 3). These results show that population density, GDP and CLP have negative impacts on ESH. Moreover, from 1995 to 2015, urbanization exerted different negative pressures on regional ecosystem health.

From 1995 to 2015, Moran’s I between GDP and ESH changed as follows: −0.237, −0.281, −0.295, −0.371, −0.168. The negative spatial correlation between GDP and ESH increased from 1995 to 2010. It decreased after reaching a peak in 2010. In 1995, Moran’s I between population urbanization (POP) and ESH was −0.366. From 1995 to 2000, the negative spatial correlation between POP and ESH decreased. It decreased from 2000 to 2015. The negative spatial correlation between CLP and ESH increased from 1995 to 2015. It reached a peak with −0.429 in 2015.

**Figure 7:**
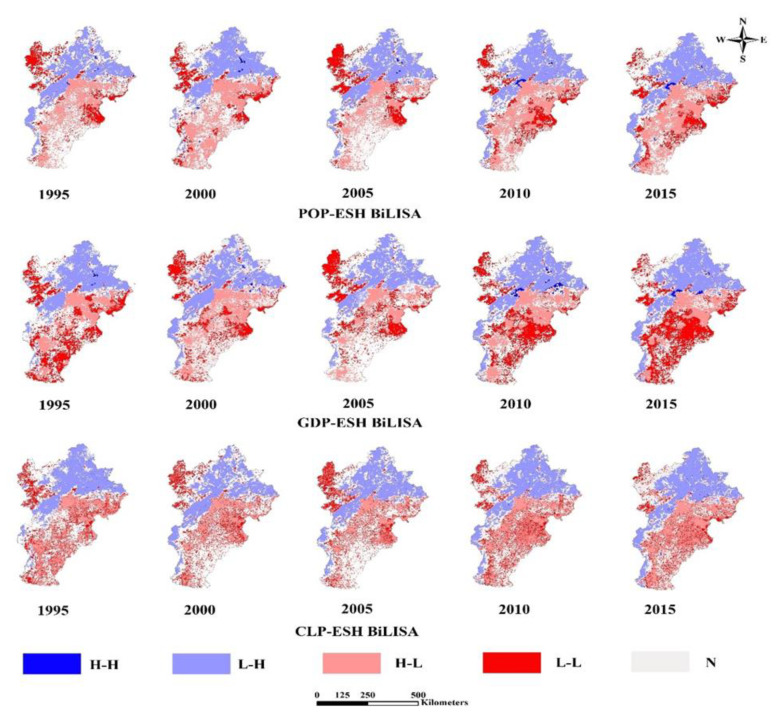
LISA cluster maps between ESH and urbanization level. (GDP: GDP urbanization; CLP: construction land proportion; POP: population urbanization; HH: high urbanization and high ESH; HL: high urbanization and low ESH; LH: low urbanization and high ESH; LL: low urbanization and low ESH).

The LISA cluster maps (Figure 7) showed four types of spatial correlations: high urbanization and high ESH (HH), high urbanization and low ESH (HL), low urbanization and high ESH (LH) and low urbanization and low ESH (LL). 

In detail, LL areas for POP and ESH distributed in the northwest and some east parts of BTH. HL areas for POP and ESH were distributed in cities, and spread adjacently with slow expansion. 

LL areas for GDP and ESH were distributed in the northwest and south parts of BTH. LL areas were shrinking in the northwest but expanding in the south. HL areas for GDP and ESH were distributed in cities, and spread adjacently with an expansion trend. 

LL areas for GLP and ESH are distributed mainly in the northwest parts of BTH. HL areas were concentrated in the surrounding areas of major cities with an expansion trend. LH areas were distributed in the north with an expansion trend.

## 4. Discussion

### 4.1. The Method of ESH Assessment and ESH Dynamics

The vigor–organization–resilience–ESV framework was applied to assess the ESH level of BTH, which helps to explain ESH dynamics more comprehensively. Involving ecosystem services in the traditional framework highlights the benefits humans obtain from ecosystems [25,29]. This method can help to study the interactions between ecosystems and society. It is meaningful for ecosystem management decision-making with emphasis of well-being in the ESH assessment [63]. 

The ESH level of BTH presents significant spatio-temporal dynamics. From 1995 to 2000, the ESH level in northern parts decreased. The ESH decrease was due to the forest land decrease. In this period, to meet the needs of urban population growth for more food and dwellings, some forest was cut down and converted to cultivated land and built-up land [30,31,32]. These activities led to NDVI and NPP decreases of the forest ecosystem. The vigor and resilience of the forest ecosystem declined. Forest patches were divided by transportation construction activities, causing a landscape connectivity decrease. The organization of the ecosystem decreased. Forest is the important contributor of ecosystem services [64], and the deforestation led to a decline in ESV. Since these indicators decreased, ESH level in BTH decreased. From 2000 to 2015, ESH dynamics of BTH showed the tendency of forming a northeast–southwest belt boundary. In the northwest of the belt, the ESH level was higher, and the improvement is obvious. In the southeast part of the belt, the ESH level was lower. The obvious differences may be related to ecological restoration projects in the BTH region. Since the late 1970s, China has conducted a series of projects to control frequent sandstorm disasters and protect ecosystems in the BTH region, such as the Beijing-Tianjin Storm Sources Control Project [65], the Grain for Green Program [66], The Taihang Mountains afforestation project [67] and the Three-North Forest Shelter Belt Program [68]. The forest and grassland area has increased from 69,120 km^2^ to 83,501 km^2^ [69]. This led to NDVI and NPP increases, improving the vigor and resilience of the ecosystems. Additionally, increased forest land and grassland helps to intercept more rainfall and reduce rainfall erosion, which alleviates the risk of regional soil erosion and sandstorm disasters [70]. It also provides more benefits (wood products, water conservation, soil retention and other ecosystem services) for humans and increases regional ESV. Since vigor, resilience and ESV have been improved, ESH of north BTH is improved due to forest land and grassland increases from these projects.

### 4.2. Urbanization Process in BTH Region

From 1995 to 2015, rapid and large-scale urbanization occurred in the BTH region. The population, economy and land urbanization all progressed. From 1995 to 2015, population density increased obviously in Beijing and Tianjin. As the development of secondary and tertiary industries in Beijing and Tianjin provided more employment opportunities, farmers moved to cities, and much of the agricultural population migrated to urban areas. Economy urbanization has developed rapidly in the BTH region. The increase of GDP is due to the increased investment in urban areas and the upgradation of the industrial structure under the guidelines from policy. The total investment in urban areas of BTH accounts for more than 85% of the total regional investment–90.60% in Beijing and 97.30% in Tianjin [30,31]. The industrial structure also changed. Under the policy’s guidance, the tertiary industries in Beijing and Tianjin urban areas have developed rapidly; the proportions have increased from 47.03% to 77.95% and 37.86% to 49.34% respectively [30,31]. Land urbanization is another considerable feature of urbanization process in BTH. From 1995 to 2015, the built-up land expanded rapidly, with a growth of 29,537.33 km^2^ [30,31,32]. The expansion of the built-up land mainly occurred in Beijing and Tianjin. Population growth and economic growth have promoted the expansion of the built-up land. In addition, this expansion is consistent with the policy of promoting urbanization [71,72,73]. 

Overall, the urbanization process of BTH gradually formed a triangular spatial pattern with Beijing, Tianjin and Tangshan as vertexes (Figure 4, Figure 5 and Figure 6). We recommend the following: (1) control population growth and promote population quality in Beijing and Tianjin; (2) adjust the industrial structure and eliminate industries with high energy consumption and high pollution; (3) control the expansion of built-up land in big cities and encourage the development of small towns in the surroundings; optimize the allocation of land and protect basic arable land and ecological land.

### 4.3. Impacts of Urbanization on ESH 

From 1995 to 2015, the level of urbanization in BTH continued to rise. The results show that urbanization had negative impacts on ESH in urban areas of BTH between 1995 and 2015. In detail, there were two stages: In 1995, POP was the dominant factor; after 1995, CLP was the dominant factor.

Urbanization affects ecosystem health in different ways. Population urbanization has a direct influence on ecosystem health through resource consumption. In BTH, residents in urban areas kept increasing year by year from 1995 to 2000, and reached a 3.26% rate of increase in 2000 [74]. To support more residents in BTH, more resources are demanded. According to the statistics, domestic water resource consumption for the urban area increased from 1677.44 million m^3^ to 1980.11 million m^3^ [75,76,77]. BTH is one of the regions where water resources are extremely scarce under rapid urbanization [78]. Additionally, it is cold and dry in winter, so high-intensity heating is required in BTH. The increased population requires more consumption of coal and natural gas in BTH for each household, which has caused more pressure on ecosystems. Along with the increase in population, economic urbanization is developing in BTH. Industry and urban commerce flourish. Advancement in the industrial output also increases resource and energy consumption. At the same time, built-up land has expanded to supply the necessary conditions for industrial production and transportation. Increases in the width and density of land exploitation activities raise spatial pressure on the ecosystems. Increased urbanization brings more disturbances to ecosystem health in cities of BTH, which tends to spread to peri-urban areas. 

It is recommended to focus not only on ecological restoration projects in the northern and northwest part of BTH to improve regional ecosystem health level, but also on the maintenance of ecosystem health in peri-urban areas. Ecosystem protection and maintenance of health levels could be achieved through reasonable strategy or policy. For BTH, an ecological function zoning can be considered a scientific strategy. In northern parts of BTH, ESH is at a strong or relatively strong level. To maintain that high level of ESH, intensive land exploitation should be prohibited. Simultaneously, ecological compensation should be conducted to coordinate the economic development and ecosystem health level maintenance. Furthermore, appropriate tourism could be developed. More efforts should be made to transform ecological advantages into economic benefits in these regions with high ecosystem health. For regions with weaker ESH, more environmental investments from local government are needed to increase the capacity of purification and self-regulation of ecosystems, which helps to reduce disturbances on the ecosystems and improve the ESH. In addition, it is expected to reduce the pressure from urbanization by controlling total pollution emissions through policy interventions and the widespread use of cleaner production technology. To achieve sustainable development of BTH, policy guidance from society is crucial.

## 5. Conclusions

In this study, we first assessed the ESH of BTH, and then we explored the correlations between urbanization level and ESH from a spatio-temporal perspective by applying multi-source data. We found that the ESH in BTH increased from 2005 to 2015. The increase of ESH in north regions was more obvious than others. Moreover, urbanization had a negative impact on ESH. The dominant factor in 1995 was POP, and Bivariate Moran’s I was −0.366; CLP was the dominant factor from 2000 to 2015, and Bivariate Moran’s I went from −0.362 to −0.429. Rapid urbanization had the greatest negative impact on ESH in urban areas. It has a trend of spreading from urban areas to adjacent areas. We suggested that ecological restoration projects should be continued in northern regions of BTH. Additionally, attention was not only paid to ecological restoration in the northern and northwest part of BTH, but also to the maintenance of ecosystem health in peri-urban areas under rapid urbanization. In the future, more indicators for the VOR-ESV framework should be considered and more efforts should be made to analyze the effects of urbanization on ecosystem structure and function. 

## Figures and Tables

**Table 1:** Framework and indicators.

Framework	Indicators	Number	P/N ^2^
Vigor	NDVI (Normalized Difference Vegetation Index)	X_1_	P
Organization	Shannon’s Diversity Index (SHDI)	X_2_	P
	Patch Density (PD)	X_3_	P
	Area-Weighted Patch Fractal Dimension (AWMPFD)	X_4_	P
	Patch Cohesion Index (COHESION)	X_5_	P
	Contagion Index (CONTAG)	X_6_	P
	Integral Index of Connectivity (IIC)	X_7_	P
Resilience	resilience value	X_8_	P
Function	Ecosystem service value (ESV) ^1^	X_9_	P

^1^ The ecosystem service value coefficients showed in Table 2. ^2^ P represents positive and N represents negative.

**Table 2:** The ecosystem service value coefficients of Beijing-Tianjin-Hebei (BTH) [50,51]. Unit: yuan/hm^2^.

Ecosystem Service		Land Use Types					
		Forest	Grassland	Water	Cropland	Built-Up Land	Unused Land
Provisioning Service	Food production	406.20	406.20	1306.91	1518.85	0.00	0.02
	Raw materials	936.03	600.47	494.51	688.78	0.00	0.02
Regulating service	Gas regulation	3090.68	2136.98	1766.10	1200.95	0.00	0.01
	Climate regulation	9254.36	5633.86	4503.56	635.80	0.00	0.09
	Waste treatment	2755.12	1854.41	9113.08	176.61	0.00	0.15
	Water regulation	6799.49	4132.67	153,014.90	565.15	0.00	0.17
Supporting service	Soil formation & protection	3761.79	2596.17	2136.98	1783.76	0.00	0.11
	nutrient cycling	282.58	194.27	158.95	211.93	0.00	0.01
	Biodiversity maintenance	3426.23	2366.57	6375.62	229.59	0.00	0.10
Cultural service	Recreation & Aesthetic value	1501.19	1042.00	4344.61	105.97	0.00	0.05

**Table 3:** Bivariate Moran’s I between ESH and GDP, POP and CLP.

Type	Year	1995	2000	2005	2010	2015
GDP	Moran’s I	−0.237	−0.281	−0.295	−0.371	−0.168
	*z*-value	−311.34	−377.1	−378.89	−481.39	−228.31
	*p*-value	0.01	0.01	0.01	0.01	0.01
POP	Moran’s I	−0.366	−0.359	−0.355	−0.276	−0.267
	*z*-value	−447.5	−445.52	−443.55	−364.02	−351.55
	*p*-value	0.01	0.01	0.01	0.01	0.01
CLP	Moran’s I	−0.351	−0.362	−0.388	−0.407	−0.429
	*z*-value	−401.83	−397.54	−426.09	−444.28	−493.85
	*p*-value	0.01	0.01	0.01	0.01	0.01

## Data Availability

Not applicable.
